# Male Choice in the Stream-Anadromous Stickleback Complex

**DOI:** 10.1371/journal.pone.0037951

**Published:** 2012-06-11

**Authors:** Jeffrey S. McKinnon, Nick Hamele, Nicole Frey, Jennifer Chou, Leia McAleavey, Jess Greene, Windi Paulson

**Affiliations:** 1 Department of Biology, East Carolina University, Greenville, North Carolina, United States of America; 2 Department of Biological Sciences, University of Wisconsin-Whitewater, Whitewater, Wisconsin, United States of America; The University of Queensland, St. Lucia, Australia

## Abstract

Studies of mating preferences and pre-mating reproductive isolation have often focused on females, but the potential importance of male preferences is increasingly appreciated. We investigated male behavior in the context of reproductive isolation between divergent anadromous and stream-resident populations of threespine stickleback, *Gasterosteus aculeatus,* using size-manipulated females of both ecotypes. Specifically, we asked if male courtship preferences are present, and if they are based on relative body size, non-size aspects of ecotype, or other traits. Because male behaviors were correlated with each other, we conducted a principal components analysis on the correlations and ran subsequent analyses on the principal components. The two male ecotypes differed in overall behavioral frequencies, with stream-resident males exhibiting consistently more vigorous and positive courtship than anadromous males, and an otherwise aggressive behavior playing a more positive role in anadromous than stream-resident courtship. We observed more vigorous courtship toward smaller females by (relatively small) stream-resident males and the reverse pattern for (relatively large) anadromous males. Thus size-assortative male courtship preferences may contribute to reproductive isolation in this system, although preferences are far from absolute. We found little indication of males responding preferentially to females of their own ecotype independent of body size.

## Introduction

Speciation research increasingly points toward divergent or disruptive ecological selection as a key cause of speciation. Such selection may drive the evolution of behavioral reproductive isolation either through pleiotropy as a byproduct of ecological adaptation or as an adaptation itself, through reinforcement or a similar process [1]–[7]. Most studies of mate choice and behavioral reproductive isolation have focused on female choice, but of late male preferences have been receiving greater attention from both theoreticians (e.g. [8], [9]; reviewed by [10]) and empiricists (e.g. [11]–[15]). Both sexes should exhibit mating preferences in a variety of contexts and male preferences are considered especially likely when males provide substantial parental care and/or females vary greatly in fecundity (e.g. [8], [16]), as in sticklebacks [17].

Here we investigate the potential role of male behavior in reproductive isolation between divergent anadromous and stream-resident populations of the threespine stickleback, *Gasterosteus aculeatus*. The stickleback species pairs are now well established as an important model system for the study of speciation [18]–[21] and of evolution generally [17], [22]. The stream-anadromous species pairs have been less extensively investigated than British Columbia’s lake pairs but, although hybridization is frequent at some sites [23]–[25], at other localities small-bodied stream-resident and large-bodied, migratory anadromous populations breed side-by-side with few phenotypically intermediate individuals observed. The absence of intermediates is due in part to behavioral reproductive isolation [18], [19], [26].

In a large-scale study, McKinnon et al. [27] found that, across continents and ocean basins, there is a parallel pattern of behavioral isolation among relatively small bodied stream-resident and relatively large bodied anadromous populations, and body size plays an important role in mediating pre-mating isolation. Moreover, manipulation of female size significantly affected patterns of reproductive isolation, with females manipulated to a size similar to that of a male partner experiencing successful courtship more often than females manipulated to a different size, independent of actual female ecotype. However, positive assortment by ecotype independent of the size manipulation was still present, if less pronounced. In the current study, we present new data on male behavior in the latter experiment in order to address mainly two questions. First, we ask if males alter their behavior in response to the female body size manipulation in a manner such that male behavior and preferences might contribute to pre-mating isolation and to the patterns of courtship success observed in that experiment. Second, we ask if males preferentially court females of the same form, anadromous or stream-resident, independent of body size. An alternative hypothesis, supported by data from the limnetic-benthic stickleback systems [14], is that males alter their behavior so as to most successfully court a given female, rather than to reduce the probability of heterotypic spawning or continued courtship–i.e., males of all forms should converge on the courtship behaviors preferred by the females of the ecotype with which they are then interacting. Beyond these focal issues, we also ask if there are consistent differences in the frequencies of the various courtship behaviors shown by males of each form. This work is noteworthy for the use of size-manipulated females of different forms and for the insights emerging from a principal component analysis of the male behavioral data.

## Materials and Methods

### Ethics Statement

All work was approved by the University of Wisconsin-Whitewater Institutional Animal Care and Use Committee (Animal Welfare Assurance A4087-01) and carried out in strict accordance with national guidelines.

The general experimental design, including collection and rearing methods, is presented elsewhere [26] and recounted only briefly here. Females were from laboratory pure crosses of each ecotype from each of the two regions: Japan and British Columbia (Salmon River). Sticklebacks from British Columbia were sympatric with the opposite ecotype whereas fish from Japan were not. To produce large (mean SL = 55.25 mm, SE = 0.937, n = 56) and small females (mean SL = 43.73 mm, SE = 0.449, n = 71) of each ecotype (and from each region), ‘large’ fish were raised to two years of age at relatively low densities whereas ‘small’ fish were raised to one year of age at relatively high densities. All fish were raised in weakly brackish water (approximately 3 ppt salinity). Males were wild-captured from the Salmon River, British Columbia. Anadromous males (mean SL = 60.93 mm, SE = 0.254, n = 60) were larger than stream males (mean SL = 47.19 mm, SE = 0.383, n = 67).

Mating trials were ‘no choice’ tests involving one male and one female paired in a 96 liter aquarium and allowed to interact freely (sample sizes for each male-female combination in Table S1). Although some males were tested with a second, different female, in analyses presented here we take the conservative approach of including only the first trial of each male, since our focus is on male behavior.

To minimize the influence of female behavior on our male courtship data, male behavior was scored only from the first five minutes of each trial [28], [29], or until the end in the few (8 of 127 total) trials that ended in under five minutes. Male behaviors were calculated per minute. The full suite of male behaviors usually recorded in stickleback courtship studies [30] was recorded but only the following subset, which do not require direct participation by the female and thus are relatively independent of female behavioral responsiveness, are included here: bite-bump–any contact of male’s head with female; zig-zag–dart first roughly away then toward female in a horizontal plane; nest work–any behavior (fanning, creeping through, boring, etc.) directed toward the nest (scored as a single bout until the male moves more than one body length from the nest or initiates a bite-bump); direct lead–male swims directly toward nest after courting female. It should be noted that one behavior, bite-bump, may also occur entirely outside the context of courtship, for example during agonistic interactions between males, or between females.

Videotapes were scored using event recorder software operating on personal digital assistants. Each tape was scored and checked by one of three individuals and all scores were checked again by a single investigator (Hamele).

We calculated events per minute for each of the behaviors then conducted log_10_ transformations (of the raw data plus one) to improve normality. Most male behaviors were correlated with each other (details in results), so we conducted a principal components analysis on the correlations and ran analyses on the first two principal components. Data were analyzed using JMP 9.0.

Where there was a clear directional prediction for a nominal term in the analysis, we used the more powerful ordered heterogeneity test following Rice and Gaines [31]. This test combines an ANOVA or a related test with Spearman’s rank correlation coefficient, the latter accounting for the direction of the prediction. We specifically tested the predictions that stream males should more vigorously court small females and anadromous males large females (i.e. females manipulated to similar sizes), and that males should more vigorously court females of the same ecotype independent of size (both after correcting for any significant main effect(s) by using residuals).

## Results

### 1. Overall Correlations among Male Behaviors

Across the complete data set (n = 127) the four male behaviors were significantly correlated with each other (Table 1) with only the exception of direct lead and bite-bump. In general, correlations with bite-bump tended to be lower than correlations among the other three variables.

**Table 1:** Correlations between log transformed male behaviors for stream and anadromous males pooled (**p<0.005; ***p<0.0001; n = 127).

Variable	Direct Lead	Nest work	Zig-Zag
Nest work	0.6248***		
Zig-Zag	0.4186***	0.6086***	
Bite-bump	0.0311	0.2887**	0.2628**

In a principal component analysis, the first principal component (PC1) accounted for 55.1% of the variance. The variables loading most strongly on PC1, as indicated by the eigenvectors, were nest work (0.606), zig-zags (0.544) and direct leads (0.510); bite-bumps showed a lower loading at 0.277. We interpret PC1 as being associated with unambiguous, vigorous courtship in which males display to the female, try to lead her to the nest to spawn, and work on the nest in preparation for spawning; because PC1 is both readily interpretable and accounted for more than 55% of variance, it is emphasized in subsequent analyses. PC2, accounting for 24.5% of variance, was much more strongly associated with bite-bumps (0.883) and negatively associated with direct leads (−0.461) and nest work (−0.066); zig-zags essentially did not load on PC2 (0.057). We interpret PC2 as characterizing either rejection of the female or a distinct, perhaps more aggressive aspect of courtship not closely associated with preparation for spawning.

### 2. Male Courtship Behavior, Male Ecotype and Female Characteristics

**Figure 1:**
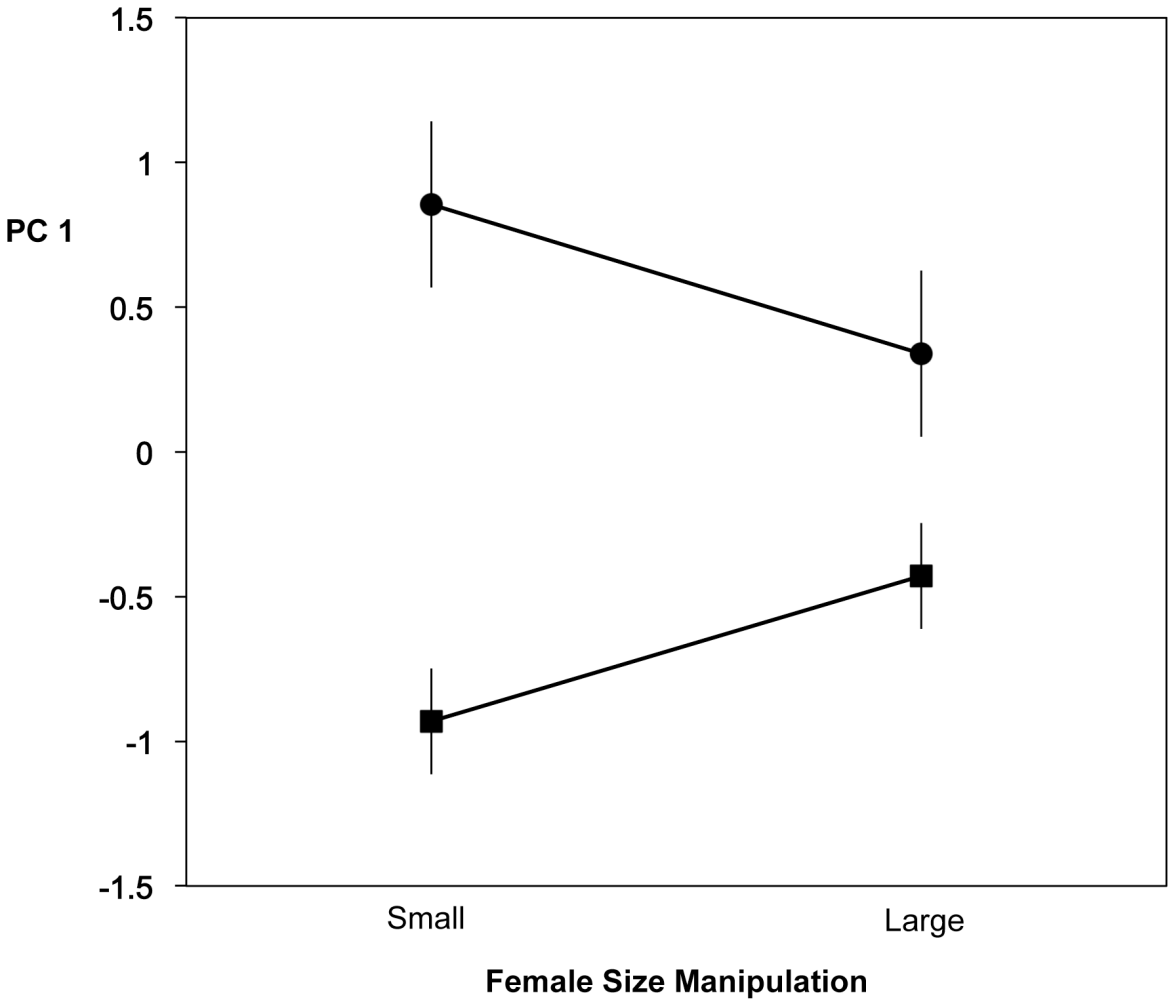
Means for PC1 of log male behaviors versus female size manipulation. Stream male data circles, anadromous male data squares. Error bars are SE’s.

We analyzed male courtship behavior using a full factorial ANOVA with the independent variables male ecotype (anadromous or stream-resident), female ecotype (anadromous or stream-resident), female region (British Columbia or Japan) and female size class (manipulated to small or large size). For the dependent variable PC1, the most significant independent variable was male ecotype (Fig. 1): stream-resident males exhibited consistently more vigorous and clearly positive courtship than did anadromous males (F = 20.923, P<0.0001, df = 1, 111). The only other significant effect was an interaction between male ecotype and female size manipulation (p<0.01 for ordered heterogeneity test following Rice and Gaines [31] and testing the prediction that stream males should more vigorously court small females and anadromous males large females: Fig. 1). Thus males courted females manipulated to a size similar to their own more vigorously.

We found no evidence of males responding preferentially to females of their own ecotype independent of body size. The male ecotype by female ecotype term was nonsignificant (p>0.2 for ordered heterogeneity test of the prediction that males should more vigorously court females of the same ecotype). Higher order interaction terms involving male and female ecotype were also all nonsignificant, as were all other terms in the ANOVA (P>0.09 in all cases).

When a measure of female preference and courtship success, whether or not the female inspected the male’s nest at some point in the full trial [27], [32], [33], was added to the model, trials with nest inspections averaged significantly higher for PC1 than did trials without an inspection (means: 0.8144, SE = 0.2213, −0.6024, SE = 0.1197, respectively; F = 27.3009, P<0.0001, df = 1, 110) whereas the pattern of preferential courtship toward similar size females was rendered nonsignificant (P>0.1, ordered heterogeneity test as above). This suggests that vigorous male courtship and mating success are closely related. The consistent differences between males of different ecotypes, with stream males generally scoring higher on PC1, remained highly significant (F = 25.9093, P<0.0001, df = 1, 110). We further asked whether the relationship between nest inspection and PC1 was consistent for the two male ecotypes, by adding to the model an interaction term for nest inspection and male ecotype; this term was nonsignificant (F = 0.9497, P = 0.3319, df = 1, 109), suggesting the relationship did not differ for the two ecotypes.

**Figure 2:**
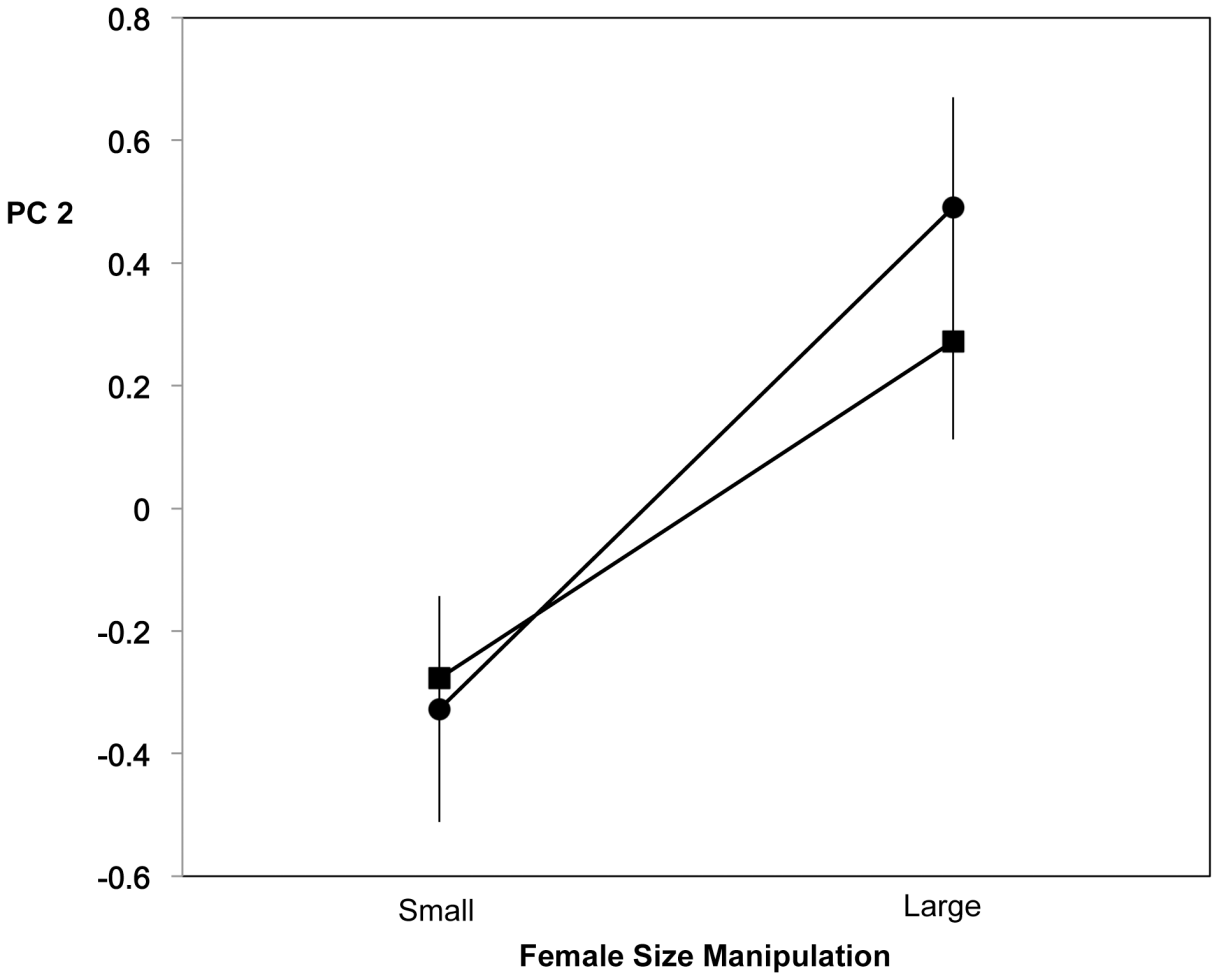
Means for PC2 of log male behaviors versus female size manipulation. Stream male data circles, anadromous male data squares.

For the second principal component (PC2), which was associated mainly with more frequent bite-bumps and less frequent leads, there was no overall difference between stream and anadromous males (Fig. 2: F = 0.2462, P = 0.6207, df = 1, 111). In contrast to results for PC1, the most significant term in the analysis was the main effect of the female size manipulation, with PC2 scores higher for males presented with larger females (Fig. 2: F = 14.9315, P = 0.0002; df = 1, 111). The male ecotype-female size class interaction was not significant (F = 0.5365, P = 0.4654, df = 1, 111; ANOVA result presented rather than ordered heterogeneity test because there is no clear prediction for PC2), indicating that males of the two ecotypes discriminated in largely the same way between large and small size class females. Two additional terms were also significant, if weaker. Anadromous females elicited higher levels of PC2 from males than did stream females (for female ecotype, F = 4.3590, P = 0.0391, df = 1, 111) and Japan females elicited higher PC2 levels than did BC females (for female region, F = 6.7709, P = 0.0105, df = 1, 111). There was again no significant interaction between male ecotype and female ecotype, suggesting no ecotype-assortative male courtship independent of body size (F = 0.0152, P = 0.9020, df = 1, 111). No other interactions were significant (P>0.46 in all cases).

**Figure 3:**
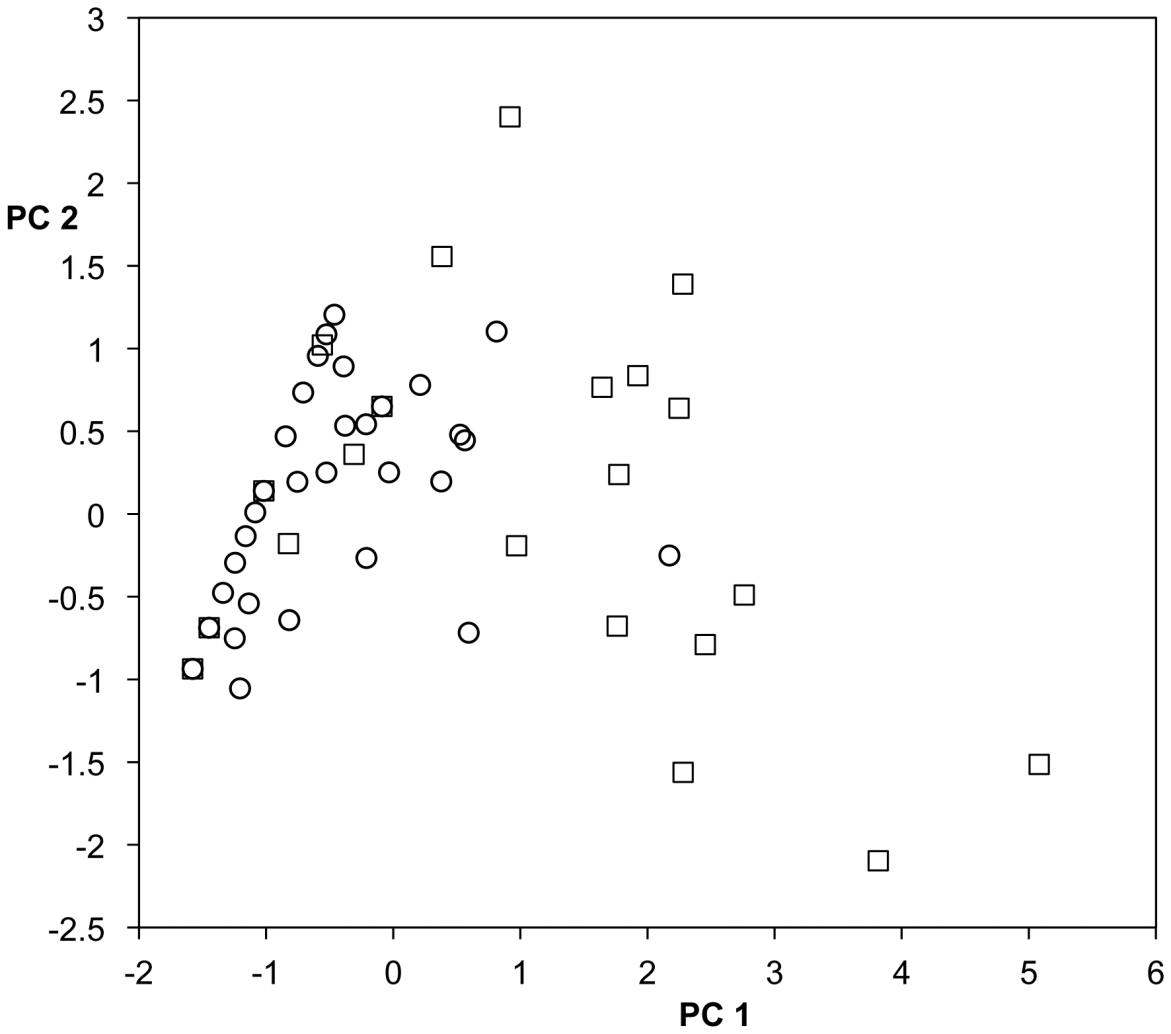
PC1 versus PC2 for anadromous males. Trials with a nest inspection, squares, trials with no nest inspection circles.

**Figure 4:**
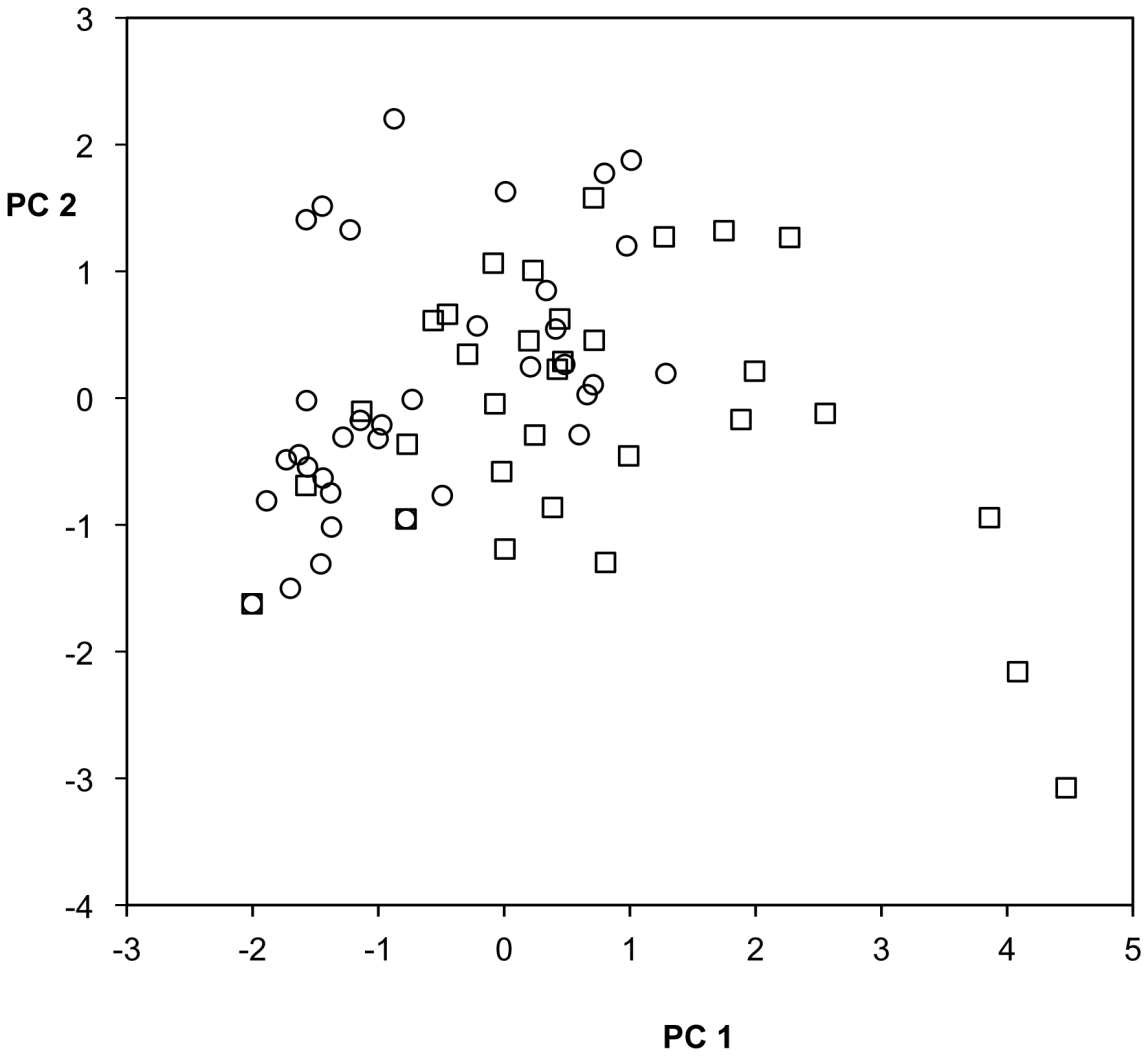
PC1 versus PC2 for stream males. Trials with a nest inspection, squares, trials with no nest inspection circles.

Nest inspection was not significant when added to the model (F = 0.0097, P = 0.9217, df = 1, 110) and all significant effects in the preceding analysis remained so, suggesting that PC2 is not so clearly closely associated with courtship success as PC1, overall (also see Figs. 3, 4). When we added to the model an interaction term for nest inspection and male ecotype, it was also nonsignificant (F = 1.5425, P = 0.2169, df = 1, 109), although the trends in the data were interesting. Anadromous males in trials with a nest inspection showed higher levels of PC2 whereas stream males showed lower levels.

To further assess the relationship between female body size and male courtship behavior for males of each ecotype, we conducted additional, somewhat simplified analyses for each principal component of the relationship between male ecotype, female body size, and their interaction. Rather than categorizing females simply as large or small based on the size manipulation, as for the ANOVA’s, this GLM is based on actual standard lengths of individual females and thus takes into account all variation in female body size (but note that the size manipulation analysis was important because it minimizes correlates of size that might result from using only natural variation). As in the previous analyses of PC1, male ecotype was highly significant (F = 33.5354, P<0.0001, df = 1, 123) owing to higher scores for stream males. Female standard length had no consistent relationship with PC1 (F = 0.2320, P = 0.6309, df = 1, 123) but the interaction between male ecotype and female standard length was significant (F = 6.5135, P = 0.0119, df = 1, 123), owing to the tendency of anadromous males to respond more strongly to larger females and stream males to respond more strongly to smaller females (as in Fig. 1), confirming the analyses above. For PC2, the effect of male ecotype was again nonsignificant (F = 1.5839, P = 0.2106, df = 1, 123) whereas the effect of female size was consistent: both male ecotypes responded more strongly to large females (as in Fig. 2; female size: F = 30.1309, P<0.0001, df = 1, 123) and the interaction between male ecotype and female size was nonsignificant (F = 1.0126, P = 0.3162, df = 1, 123), again confirming the earlier analyses; this result was not affected by the inclusion of the additional significant main effects (female ecotype, region) of the earlier ANOVA’s.

### 3. Correlations among Male Behaviors by Population and the Interpretation of Patterns in Male Courtship

In an effort to more fully elucidate the different relationships between female size class and male behaviors summarized by PC1 and PC2, we analyzed the correlations among male behaviors separately for males of each ecotype (Table 2, Table 3) as well as the principal components (Table 4, Table 5; Figs. 3, 4). The main difference was for the relationships between bite-bump and other behaviors. For anadromous males, bite-bump was approximately as strongly, and significantly, correlated with the other male behaviors as those behaviors were with each other (Table 2); moreover, all four behaviors loaded strongly and similarly on anadromous male PC1 (Table 5). However, while zig-zags, direct leads and nest work were also highly correlated for stream males, none of them was significantly correlated with bite-bump (Table 3) and, as in the pooled analysis (but to a greater degree), bite-bump loaded weakly on stream male PC1 relative to the other behaviors (Table 5). Finally, in separate analyses for each male ecotype, analogous to those described above that include nest inspection, there was again a highly significant relationship between nest inspection and male ecotype-specific PC1’s (P<0.0005 in each case: Figs. 3, 4). Eigenvectors associated with PC2 are broadly similar for the two morphs, except that for anadromous males behaviors other than bite-bump tend to load more negatively on PC2. As before, PC2 is not significantly associated with nest inspection, for either ecotype (P>0.41 in both cases: Figs. 3, 4).

**Table 2:** Correlations between log transformed male behaviors for anadromous males (*p<0.05; **p<0.01; ***p<0.0005; n = 60).

Variable	Direct Lead	Nest work	Zig-Zag
Nest work	0.5335***		
Zig-Zag	0.3485**	0.5303***	
Bite-bump	0.2750*	0.4469***	0.3099*

**Table 3:** Correlations between log transformed male behaviors for stream males (**p<0.01; ***p<0.0005; n = 67).

Variable	Direct Lead	Nest work	Zig-Zag
Nest work	0.6642***		
Zig-Zag	0.3627**	0.5431***	
Bite-bump	−0.0710	0.1279	0.1862

**Table 4:** Principal component eigenvalues calculated separately for stream and anadromous males.

	Stream PC1	Anadromous PC1	Stream PC2	Anadromous PC2
Eigenvalue	2.074	2.240	1.063	0.734
Percent	51.85	55.99	26.59	18.36

**Table 5:** Principal component eigenvectors calculated separately for stream and anadromous males (all variables log transformed).

	Stream PC1	Anadromous PC1	Stream PC2	Anadromous PC2
Bite-bump	0.129	0.438	0.910	0.841
Direct lead	0.558	0.484	−0.353	−0.512
Nest work	0.627	0.576	−0.052	−0.077
Zig-zag	0.528	0.492	0.211	−0.155

For bite-bump, the most anomalous behavior in these analyses, we also conducted univariate analyses of its relationships (log-transformed, as previously noted) with female body size and nest inspection, for each male ecotype. In ANOVA’s analogous to those above but conducted separately for each male ecotype (i.e. including the terms female ecotype, female region, female size class, and all interactions), the effect of female size class was consistent for both male ecotypes, with males bite-bumping large females more frequently in both cases (Anadromous males: F = 5.6858, P = 0.0208, df = 1, 52; Stream males: F = 6.8127, P = 0.0115, df = 1, 59); all interaction terms were non-significant for both male ecotypes (P>0.2 in all cases), so the effect of female size class was not complicated by other variables. When nest inspection was added to the analyses, its effect was significant for anadromous males, who bite-bumped at a higher frequency in trials that resulted in a nest inspection (F = 5.0714, P = 0.0287, df = 1, 51); moreover, the effect of size class was rendered non-significant (F = 3.2914, P = 0.0755, df = 1, 51) with inclusion of nest inspection, indicating a strong link between female size class and nest inspection in this context. In contrast, the nest inspection term was non-significant when added to the analysis for stream males (F = 2.8190, P = 0.0985, df = 1, 58), whereas the female size class term remained significant (F = 9.2053, P = 0.0036, df = 1, 58).

Thus anadromous males who are zig-zagging, readying their nest for the female and attempting to lead her to their nest are also likely to be frequently bite-bumping her; however there is no relationship between such vigorous, successful courtship and bite-bumps for stream males. Consequently, the increase in pooled analysis PC2 toward large females may occur for different reasons in stream and anadromous males, given that it is dominated by the bite-bump behavior.

## Discussion

Male sticklebacks in this study showed more vigorous courtship toward females that were manipulated to be similar to them in body size than to females manipulated to be different in size. Because courtship was assayed early in each experimental trial, the influence of female behavior on males should have been limited. While male preferences could potentially have been based on correlated aspects of female phenotype, correlations between size and other traits should have been minimized through the size-manipulation of females from four different populations. Raising fish on similar diets and in generally similar conditions may also have minimized differences in phenotypically plastic traits.

The preferential courtship of relatively large females by anadromous males is not surprising given previous studies [34], [35] and the obvious advantage to be gained from responding strongly to females carrying large clutches. However, the failure of stream males to respond more vigorously to larger females is noteworthy, in light of the larger clutches expected of such females. Because stream males were mainly presented with females of roughly their own size or larger and anadromous males were mainly presented with females of their own size or smaller, we cannot be certain that the two types of males would show different preference functions if each were presented with females both much larger and much smaller than themselves (this is also important because males are usually smaller than females). However, they clearly did respond differently to the range of females with which they were presented in this experiment.

The superficially similar elevated behavioral response of both stream and anadromous males to large size-manipulated females, in terms of the second (pooled data) behavioral principal component, PC2, is of interest given that it may have evolved for different reasons in each ecotype and reflect basic differences in courtship behavior. Based on the supplementary analyses exclusively of anadromous male behavior, it appears that when anadromous males pursue a courtship destined to be successful, and presumably reflective of strong motivation to spawn, they include a high frequency of bite-bump; this behavior can also be aggressive but appears to be a typical part of anadromous courtship (also see [36], [37]). Thus the strong pooled PC2 response of anadromous males to large females is consistent with their strong PC1 response to such females, and appears to indicate motivation to spawn (although the trend for successful anadromous males to score higher on PC2 was not significant).

Conversely, the high pooled PC2 scores of stream males toward large females contrast with their lower scores toward such females on PC1. For stream males, the supplementary ecotype-specific analyses suggest that bite-bumps, which load heavily only on PC2 and do not correlate positively with other behaviors, are not associated with vigorous, positive courtship. Thus high PC2 scores for stream males, either in the pooled or ecotype-specific analysis, may represent aggressive rejection, or possibly very tentative courtship, of large females. The trend toward an association for stream males between failed courtship and pooled PC2 was not significant but certainly there was no positive correlation with courtship success.

Our results support and extend the findings of earlier studies of male courtship in sticklebacks from the Salmon River and the nearby Little Campbell River. In previous experiments involving paired female presentations, Salmon River stream males from the area of sympatry with anadromous sticklebacks courted and spawned with relatively small stream females preferentially, whereas males from an upstream allopatric site spawned more often with large females. Moreover, the sympatric stream males showed more frequent aggressive behavior and reduced zig-zagging toward relatively large females [18], [38]. In the Little Campbell River, courtship by anadromous males tended to be more aggressive in nature, with relatively more biting and less zig-zagging, especially early in courtship [36]. Female size was not manipulated in those studies, in contrast to the present work, and behavior was generally examined in a univariate context.

Based on data from experiments with limnetic and benthic sticklebacks, Kozak et al. [14] concluded that male sticklebacks modify their courtship to match that characteristic of a prospective female mate’s population–i.e. limnetic males court more like benthics when confronted with benthic females. Such an interpretation appears less appropriate for our results, mainly because anadromous males scored higher with large females on both principal components. Stream males exhibited relatively lower levels of PC1, which is associated with courtship success, when interacting with large females. This result is not expected if the male ecotypes were converging in their courtship behavior. In addition, stream males exhibiting higher scores on PC2, which reflects a more aggressive mode of courtship behavior, tended to experience lower courtship success, the reverse of the trend for anadromous males. It must be noted, however, that both male types scored higher on PC2 when interacting with an anadromous female, suggesting either that stream males are recognizing anadromous females to some degree independently of body size and rejecting them, or that some aspect of the anadromous female phenotype elicits a more aggressive courtship style, even if ineffectually in terms of courtship success.

Stream males responding less positively to large females in nature would tend to mate assortatively by ecotype, since anadromous females are larger. This preference may have arisen as a result of either reinforcement or direct selection, since the populations of the males tested here are sympatric. It is also possible that this preference is a byproduct of divergence in body size or some other trait, but given the benefits of fathering the large clutches produced by large females and the fact that body size differences were not extreme (compared, for example, to the differences between some populations in the large-scale comparative analyses in [27]), a strictly byproduct scenario for male preference evolution seems incomplete. We have no data that directly address the likelihood of reinforcement versus direct selection, but female egg cannibalism is known from Eastern Pacific marine/anadromous sticklebacks (e.g. [39]). Consequently, large anadromous females could present a threat to the eggs and nests of relatively small stream males, and potentially more of a threat than they present to the larger anadromous males. This threat may be still greater if stream-resident males do not experience cannibalism from stream females, and lack some defenses possessed by anadromous males. We are not aware of data on the presence or absence of female egg cannibalism in Salmon River stream females, but the relatively conspicuous, incautious courtship of stream males, relative to co-occurring anadromous males, is reminiscent of the courtship of limnetic male sticklebacks in the lake pair systems. In those systems, only benthic females are cannibals and limnetic males may court them less than they do the smaller limnetic females [29]. Reinforcement is also possible given the demonstrations of ecological inferiority of hybrids in other stickleback pairs (e.g. [40]) and the apparent selection on multiple traits when sticklebacks colonize freshwater (e.g. [19], [21], [41], [42]).

The present data sets and analyses are not designed to enable powerful assessment of the relative contributions of male versus female preferences to patterns of reproductive isolation in our study populations. But the strong relationships between male courtship form and male vigor with courtship success (specifically nest inspection), together with earlier results on spawning success of Salmon River sticklebacks in choice tests [38], at least raise the possibility that male preferences contribute to spawning patterns in stream-anadromous systems, and possibly in the comparative study that complemented these manipulations [27]. Based on combined analyses of male preferences, female preferences and spawning success, Kozak et al. [14] concluded that male preferences contribute little to patterns of reproductive isolation in limnetic-benthic systems; however, they also did not see the apparent male preferences for similar sized size females that we observed. In any case, our data do not suggest that male preferences by themselves would lead to strong reproductive isolation since both male ecotypes clearly did court both relatively large and relatively small females and differences in courtship intensity were not extreme.

Imprinting has recently been shown to play a role in female preference development, and assortative mating (or lack thereof) in benthic-limnetic pairs ([33], but see [32]). Females in the present experiment were raised artificially without exposure to their fathers so they could not have imprinted through the mechanism described in [33], although sibling effects could have been present [43]. There may have been imprinting by the wild-caught males but males did not imprint in the study by Kozak et al. [33], although they also did not find any evidence of male preferences.

In conclusion, our results suggest that stream-resident and anadromous male sticklebacks, from a site where the forms are sympatric, preferentially court females relatively similar in size to themselves when correlations between size and other traits have been minimized through a manipulative experiment. Because male courtship differs between the ecotypes, however, interpreting patterns in male behavior is not trivial. These preferences may contribute to reproductive isolation in a natural setting but at present we can draw no strong conclusions as to which evolutionary forces are responsible for male size-preferences.

## Supporting Information

Table S1 **Number of trials of each male-female combination.**
(DOC)
