# Supplementary material for: Male Choice in the Stream-Anadromous Stickleback Complex
Source: PLoS One. 2012 Jun 11;7(6):e37951. doi: 10.1371/journal.pone.0037951 (PMC3372497; doi:10.1371/journal.pone.0037951)
Supplement: Table S1 — Number of trials of each male-female combination. (DOC) [file pone.0037951.s001.doc]

| **Female Characteristics** |  |  | **Male Ecotype** |  |
| --- | --- | --- | --- | --- |
| **Female Size Class** | **Female Ecotype** | **Female Region** | **Anadromous** | **Stream** |
| Small | Anadromous | BC | 10 | 12 |
| Small | Anadromous | Japan | 10 | 10 |
| Small | Stream | BC | 10 | 10 |
| Small | Stream | Japan | 3 | 6 |
| Large | Anadromous | BC | 11 | 9 |
| Large | Anadromous | Japan | 5 | 3 |
| Large | Stream | BC | 10 | 10 |
| Large | Stream | Japan | 1 | 7 |
